# Optimal, minimax and admissible two-stage design for phase II oncology clinical trials

**DOI:** 10.1186/s12874-020-01017-8

**Published:** 2020-05-20

**Authors:** Fei Qin, Jingwei Wu, Feng Chen, Yongyue Wei, Yang Zhao, Zhiwei Jiang, Jianling Bai, Hao Yu

**Affiliations:** 1 Department of Epidemiology and Biostatistics, Arnold School of Public Health, University of South Carolina, Columbia, SC USA; 2 Department of Biostatistics, School of Public Health, Nanjing Medical University, SPH Building Room 418, 101 Longmian Avenue, Nanjing, 211166 Jiangsu China; 3 Department of Epidemiology and Biostatistics, College of Public Health, Temple University, Philadelphia, PA USA; 4 Beijing KeyTech Statistical Consulting Co., Ltd, Beijing, China

**Keywords:** Optimal design, Minimax design, Admissible design

## Abstract

**Background:**

The article aims to compare the efficiency of minimax, optimal and admissible criteria in Simon’s and Fleming’s two-stage design.

**Methods:**

Three parameter settings (*p*_1_-*p*_0_ = 0.25–0.05, 0.30–0.10, 0.50–0.30) are designed to compare the maximum sample size, the critical values and the expected sample size for minimax, optimal and admissible designs. Type I & II error constraints (*α*, *β*) vary across (0.10, 0.10), (0.05, 0.20) and (0.05, 0.10), respectively.

**Results:**

In both Simon’s and Fleming’s two-stage designs, the maximum sample size of admissible design is smaller than optimal design but larger than minimax design. Meanwhile, the expected samples size of admissible design is smaller than minimax design but larger than optimal design. Mostly, the maximum sample size and expected sample size in Fleming’s designs are considerably smaller than that of Simon’s designs.

**Conclusions:**

Whenever (*p*_0_, *p*_1_) is pre-specified, it is better to explore in the range of probability *q*, based on relative importance between maximum sample size and expected sample size, and determine which design to choose. When *q* is unknown, optimal design may be more favorable for drugs with limited efficacy. Contrarily, minimax design is recommended if treatment demonstrates impressive efficacy.

## Background

Phase II clinical trials are carried out to provide preliminary efficacy assessments of a new drug or therapy. In clinical research, phase II trials are inevitably essential for drug/therapeutic developments. They act as screening tools to discontinue ineffective drugs or warrant promising new drugs for future evaluation. Phase II trials typically employ various dosages to evaluate efficacy and safety in patients with malignant tumors. Therefore, researchers could design phase II trials to target at sensitive cancer, delimit a safety range of dosing, and outline appropriate administrations. In this sense, phase II trials may provide supportive evidence to conduct phase III trials.

Merits have been discussed in detail by Gan and Grothey [1] concerning single-arm phase II (SA-II) trials vs. randomized phase II (RP-II) trials (include both experimental and standard therapy arms). SA-II trials are found to be more preferable for single agents with tumor response end points. One of the frequently used designs in phase II cancer clinical trials is single-arm two-stage design proposed by Simon in 1989 [2]. Simon’s design has been proved to be a compelling method in initial efficacy evaluation. Based on the ethical requirement [1], once efficacy of a drug/treatment does not reach the predefined criterion in a proof-of-concept trial, the experiment will be terminated for futility to avoid more individuals accepting ineffective treatment.

One of the important advantages of single-arm phase II trials is that they involve much smaller sample size than their randomized phase II counterparts. Therefore, single-arm trials always require less time to complete and less resources invested [3]. Several studies, aiming to improve single-arm phase II clinical trials, have been employed in recent years. Shan et al. utilized results in first stage to help calculate the second stage sample size [4]. Besides, they also proposed to construct one-sided lower limits for analyzing data in adaptive phase II trials [5]. Jung and Sargent first attempted to adopt Fisher’s exact design in randomizing phase II trials [6]. Khan et al. proposed to control sample size by slightly relaxing type I error [7]. Among these, a single-arm multi-stage testing procedure, proposed by Fleming [8], is appealing. He suggested to early stop the experiment when the intermediate results are extreme, either in favor of efficacy or futility. Compared to Simon’s design, early acceptance of the drug is permitted here.

Although progression free survival is regularly used in early oncology trials, the proportion of patients whose tumors marked shrinkage is also considered as an important metric in most phase II trials [9]. Amongst all two-stage trials with dichotomous endpoints, there are many designs satisfying a type I and II error constraint, given both the upper boundary to stop the trial and lower boundary to continue the trial. Thus, Simon proposed two criteria (minimax, optimal) to estimate sample sizes. Minimax design mainly aims to minimize the maximum sample size. Alternatively, optimal design aims to minimize the expected sample size. Shuster and Mander and Thompson further extended two Simon’s criteria in their optimal designs that allow early stopping for efficacy [10, 11]. However, one limitation of two Simon’s designs is that the minimax and optimal designs may result in highly divergent sample size requirements. Based on a Bayesian decision-theoretic criterion, Jung et al. proposed a family of admissible designs that are compromises between the two Simon’s designs [12, 13].

This article attempts to systematically compare minimax, optimal and admissible criterions in both Simon and Fleming’s two-stage designs. The rest of the paper is arranged as follows. In section 2, the conception of Simon’s optimal and minimax two-stage design, as well as Fleming’s two-stage design and Jung’s admissible design are reviewed. In section 3, a variety of design parameters are used to illustrate estimated sample sizes based on three criterions in both Simon and Fleming’s two-stage designs. In section 4, a practical example is adopted to help explain the merits of Simon’s two-stage design, Fleming’s two-stage design and admissible design. In section 5, the recommendations and implementations of optimal, minimax and admissible design are discussed.

## Methods

Consider a single-arm design with tumor response rate as the primary endpoint, where a binary outcome is defined as either “response” or “no response”. We want to test the hypotheses:

$$ {H}_0:p\le {p}_0 vs.\kern0.5em {H}_1:p>{p}_0 $$

with type I error rate *α* and type II error rate *β*. Here *p* denotes the true response rate, *p*_0_ is a fixed value that denotes the maximum response probability in order to terminate trial early. In practice, we will define *p* = *p*_1_ in the alternative hypothesis to represent the minimum response probability to warrant further studies in subsequent trials, therefore, the power of the test will be calculated at *p = p*_1_ > *p*_0_. If the null hypothesis is rejected, the study will be extended to phase III stage, given the warranted therapeutic efficacy. Otherwise, the study will be terminated, given the insufficiently promising efficacy.

### Simon’s two-stage design

**Fig. 1:**
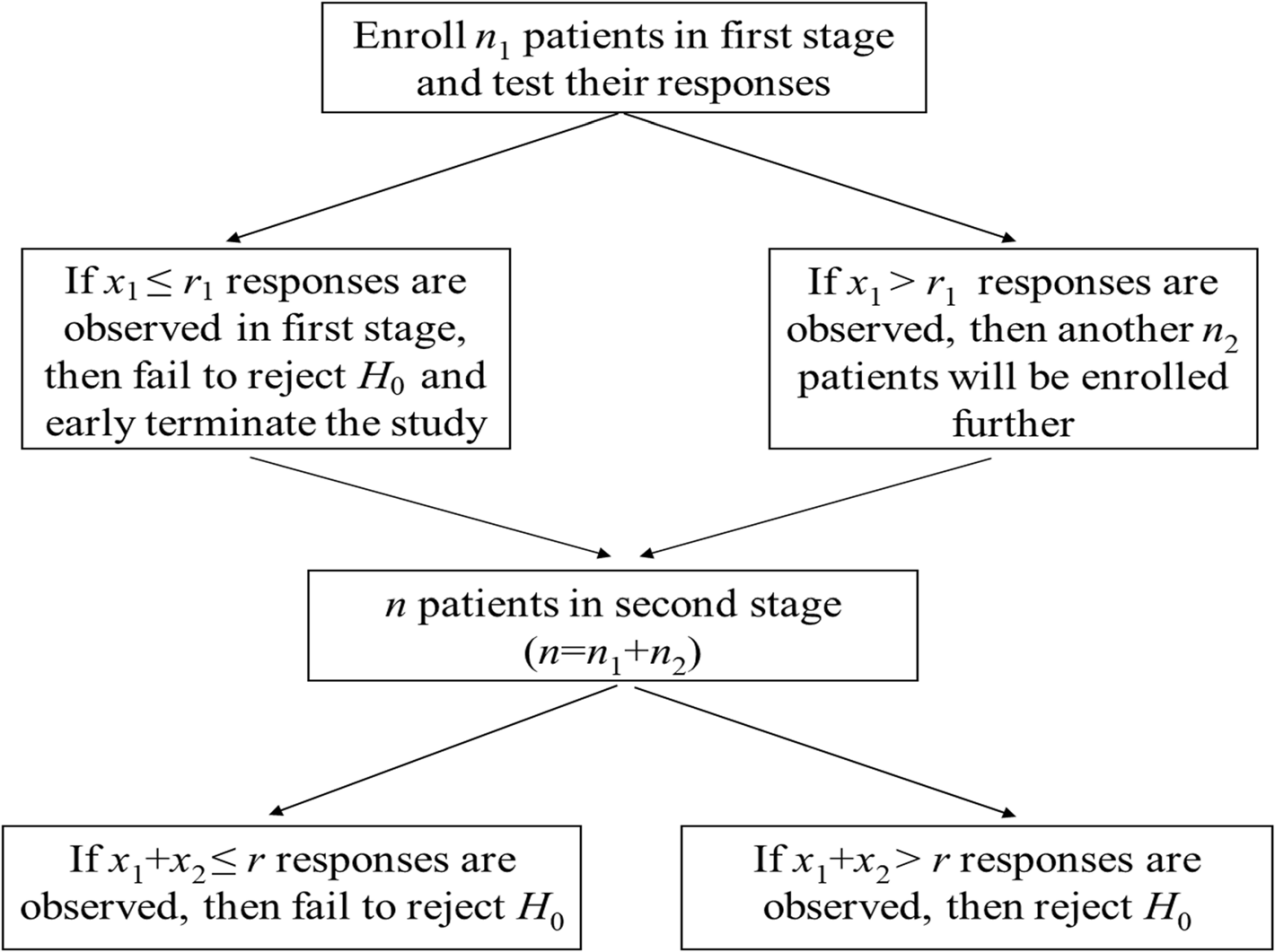
Flowchart for Simon’s two-stage design

A most widely used two-stage design is proposed by Simon [2]. Two different two-stage designs are introduced that allow early trial termination for futility. Details are illustrated in Fig. 1. In the figure, we define.

*n*_1_, *n*_2_: the number of subjects in the first and second stage, respectively, and *n* = *n*_1_ + *n*_2_;

*x*_1_, *x*_2_: the number of responses observed in the first and second stage, respectively;

*r*_1_, *r*: the number of rejection points (under *H*_0_) in the first and second stage, respectively.

Thus, the probability of early termination (*PET*) at the end of first stage (under null hypothesis) is

$$ {PET}_{\mathrm{S}1}=B\left({r}_1;{n}_1,{p}_0\right) $$

where suffix S is used to represent the result of Simon’s design. Consequently, the probability of rejecting the treatment is

$$ {P}_{\mathrm{S}}(R)={PET}_{\mathrm{S}1}+\sum \limits_{x={r}_1+1}^{\min \left({n}_1,r\right)}b\left(x;p,{n}_1\right)B\left(r-x;p,{n}_2\right) $$

Here *b*(*x;p,n*) and *B*(*x;p,n*) are the probability mass and cumulative binomial distribution function, respectively [14].

For any pre-fixed values of *p*_0_, *p*_1_, *α*, and *β*, we can enumerate the candidate designs with different (*n*_1_, *PET*_S1_, *EN*) combinations, where *EN* is the expected sample size,., i.e.,

$$ {EN}_{\mathrm{S}}={n}_1+\left(1-{PET}_{\mathrm{S}1}\right){n}_2 $$

An optimal design is considered to minimize the expected sample size. Alternatively, a minimax design minimizes the maximum sample size *n* = *n*_1_ + *n*_2_, amongst these candidates designs. If there is more than one single candidate design with smallest *n*, the one with the smallest *EN*_S_ (under null hypothesis) is chosen within all the possible minimax designs. 

### Fleming’s two-stage design

**Fig. 2:**
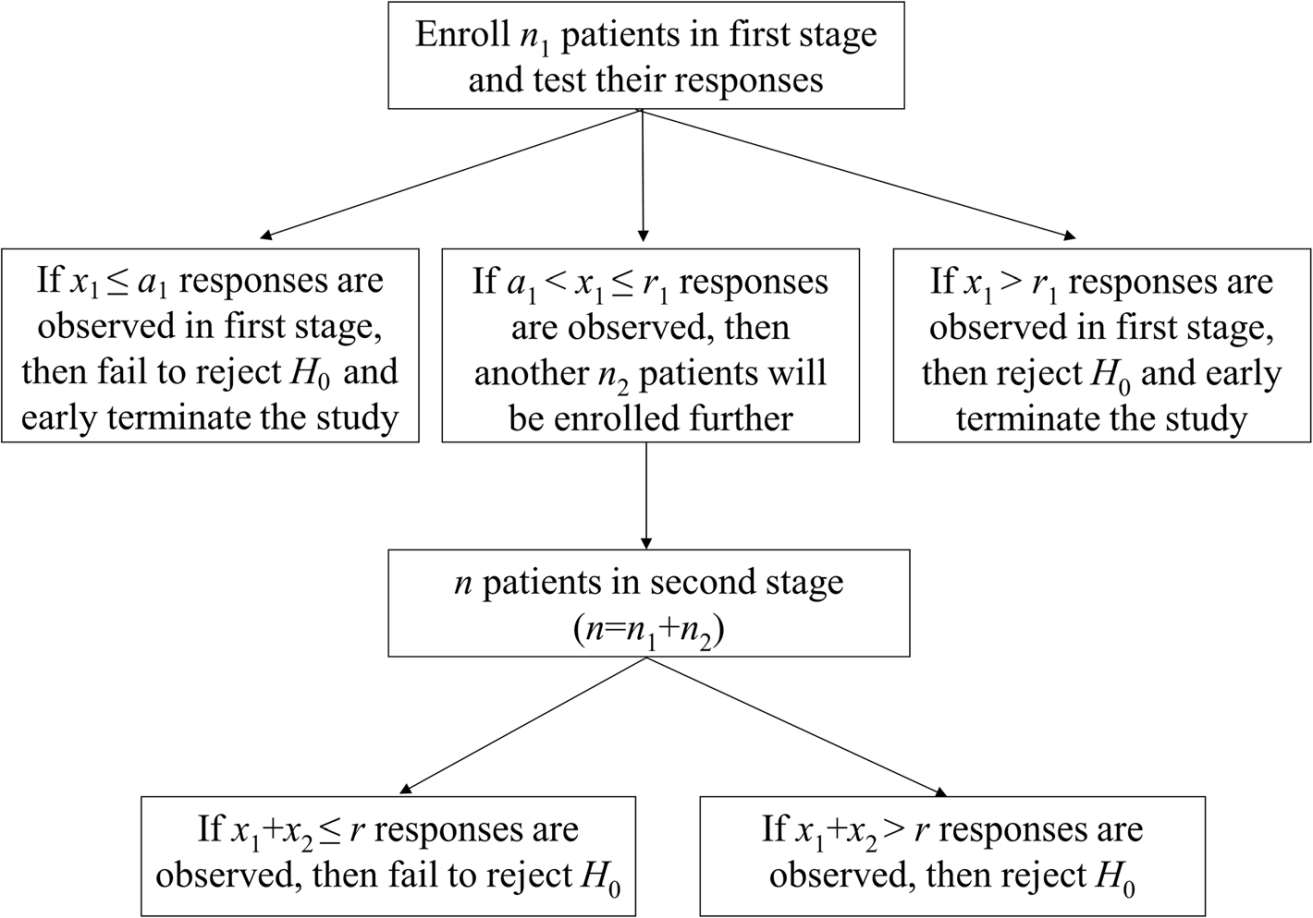
Flowchart for Fleming’s two-stage design

Unlike Simon’s two-stage design, Fleming’s design additionally allows early trial termination due to high successful response rate [8]. In Fleming’s two-stage design, one more character, *a*_1_, is required and it denotes a threshold of acceptance point (under *H*_0_) in the first stage. A single-arm two stage trial with both futility (*a*_1_) and superiority (*r*_1_) values in the first stage and a rejection value (*r*) in the second stage are described in Fig. 2.

Based on Fleming’s two-stage design, the probability of rejecting the treatment is

$$ {P}_{\mathrm{F}}(R)=B\left({a}_1;{n}_1,{p}_0\right)+\sum \limits_{x={a}_1+1}^{r_1}b\left(x;{n}_1,{p}_0\right)B\left(r-x;n-{n}_1,{p}_0\right) $$

where suffix F is used to represent the results of Fleming’s design [14]. The probability of early termination (*PET*) at the end of first stage (under *H*_0_) is

$$ {PET}_{\mathrm{F}1}=B\left({a}_1;{n}_1,{p}_0\right)+\left(1-B\left({r}_1;{n}_1,{p}_0\right)\right) $$

Thus, the expected sample size (*EN*) is

$$ {EN}_{\mathrm{F}}={n}_1+\left(1-{PET}_{\mathrm{F}1}\right){n}_2 $$

Although Fleming’s design ensures sample sizes no larger than the single-stage design, a limitation is that calculated critical values for accepting and rejecting the null hypothesis are based on pre-fixed sample sizes at stage 1 (*n*_1_) and stage 2 (*n*_2_), which may be undesirable for investigating and planning optimal designs. To remedy, Mander and Thompson extended Simon’s optimal and minimax criteria in Fleming’s two-stage design [10]. Such design will benefit from stopping early for either futility or efficacy, while preserve its simplicity and the small sample size.

### Admissible design

Very often, the minimax design has a much smaller maximum sample size *n* than that of the optimal design, but it has an excessively large expected sample size *EN*. Similarly, optimal design requires a much smaller *EN*, but it suffers a considerably larger *n* as compares to the minimax design. In planning a phase II trial, we usually find ourselves in a dilemma when we must consider choosing one of the two designs by comparing the expected sample size and maximum sample size.

To overcome, it is desirable to search for a design between the optimal design and the minimax design such that it has *EN* close to that of the optimal design and *n* close to that of the minimax design. Jung et al. proposed an admissible adaptive design based on a Bayesian decision-theoretic criterion to compromise between *EN* and *n* [12, 13]. A design is called candidate design if it minimizes *EN* for a given *n* while satisfying the (*α*, *β*)-constraint. For pre-specified (*p*_0_, *p*_1_, *α*, *β*), let *R* denotes the space of all candidate designs satisfying the (*α*, *β*)-constraint, with *n* no more than an achievable accrued number of subjects *N* during the study period. For any given design *d* ∈ *R*, we consider its two outcomes *n*(*d*) in minimax design or *EN*(*d*) in optimal design. Let *Q* be a probability distribution defined over {*n*(*d*), *EN*(*d*)} as *Q*(*n*(*d*)) = *q* and *Q*(*EN*(*d*)) = 1-*q*, where *q* ∈ [0, 1]*.*

Thus, for any design *d* ∈ *R*, the expected loss can be defined as

$$ \rho \left(q,d\right)=q\times n(d)+\left(1-q\right)\times EN(d), $$

and the Bayes risk is defined as

$$ {\rho}^{\ast}\left(\rho, d\right)={\displaystyle \begin{array}{c}\mathit{\operatorname{inf}}\kern0.5em \rho \left(q,d\right)\\ {}d\in R\end{array}} $$

Any design *d* ∈ *R* whose risk equals to the Bayes risk would be regarded as Bayes design, which will then be defined as admissible design against distribution *Q*. Note that *q* ∈ [0, 1] reflects the relative importance between maximum sample size and expected sample size in designing a phase II study. Thus, the minimax design is a special Bayes design with *q* = 1 and optimal design is a special Bayes design with *q* = 0. Conversely, for any *q* ∈ [0, 1], if no Bayes risk fits any design *d* ∈ *R*, the design would be defined as inadmissible.

Jung et al. [13] firstly proposed to apply admissible design to Simon’s two-stage design. In this article, we extend such admissible design to Fleming’s two-stage design, too.

## Results

To compare the performance of optimal, minimax, and admissible design in Simon’s and Fleming’s two-stage design, the effect difference “*p*_1_-*p*_0_” are set to be 0.2 for *p*_0_ = 0.05, 0.10 and 0.30, and type I & II error constraints “(*α*, *β*)” vary across (0.10, 0.10), (0.05, 0.20) and (0.05, 0.10), respectively. These values are appeared in both Simon’s and Fleming’s two-stage design papers and are more representative to show sufficient promise to justify a definitive evaluation [15–17]. We calculate the true type I error and power (*α*_T_, 1-*β*_T_), sample size required in the first stage (*n*_1_), threshold values (*a*_1_, *r*_1_) for early termination, *PET*_1_, maximum sample size (*n*), threshold value (*r*) in the second stage, *EN* and the probability range (*q*) when each design is regarded as a good Bayes design.

**Fig. 3:**
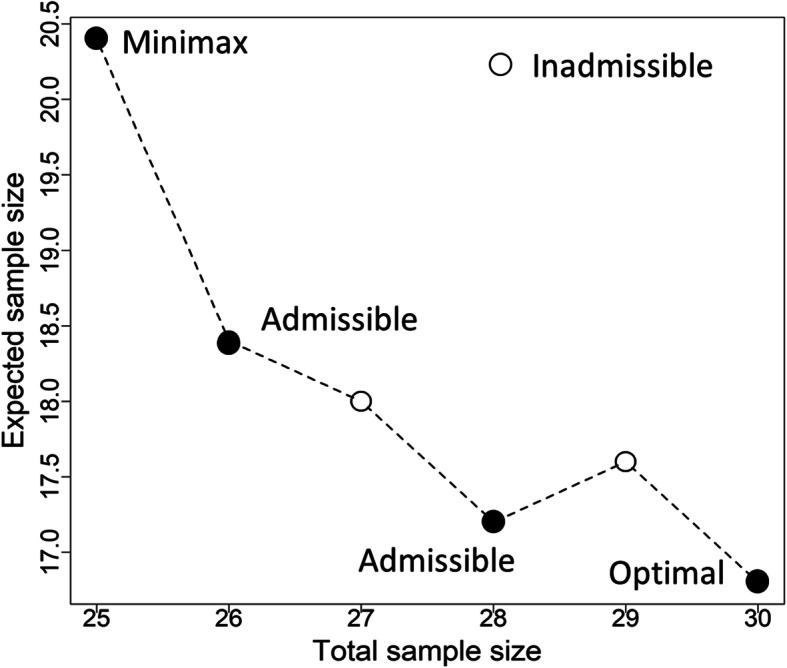
Minimax, admissible and optimal design for (*p*_0_, *p*_1_, *α*, *β*) = (0.05, 0.25, 0.05, 0.10) based on Simon’s design

Based on Simon’s two-stage design, Table 1 displays the optimal, minimax and admissible designs with pre-specified design parameters under *H*_0_. For each parameter setting of (*p*_0_, *p*_1_) and (*α*, *β*), the *EN* is much smaller than *n*. It is not difficult to find that the maximum sample size *n* of admissible design is smaller than optimal design but larger than minimax. Meanwhile, the expected samples size *EN* of admissible design is smaller than minimax design but larger than optimal design. Taking (*p*_0_, *p*_1_, *α*, *β*) = (0.05, 0.25, 0.05, 0.10) for example. In optimal design, the number of subjects required in the first stage is 9. Trials will be early terminated if no more than one response is seen in this stage. Otherwise another 21 subjects would be further enrolled, thus the maximum sample size reaches 30 at the end of the trial. The expected sample size is 16.8 and the probability of early termination is 0.630. Two admissible designs are given here, where *n* and *EN* are (28, 17.2) when *q* lies between 0.167 ~ 0.375, and (26, 18.4) when *q* lies between 0.375 ~ 0.667, respectively. For minimax design, the required maximum sample size is 25, which is five fewer than that of optimal design; while the expected sample size is 20.4, which is obviously larger than that of optimal design. A plot of *EN* against maximum sample size under this setting is illustrated in Fig. 3. The first and last dots are minimax and optimal design, respectively. Two identified Bayes candidate designs within this range are marked as “admissible”. Note, however, that some candidate designs (second and fourth design under (*p*_0_, *p*_1_, *α*, *β*) = (0.05, 0.25, 0.05, 0.10)) cannot reach Bayes risk, since their loss functions are not competitive (cannot get smaller value) over other designs for any value of *q* between [0, 1]. Such designs are symbolized as “inadmissible” in our study. In other words, such “in admissible” design may NOT be regarded as a good one according to a Bayesian decision-theoretic criterion, even though both sample size and *EN* are still deterministic given (*p*_0_, *p*_1_, *α*, *β*).

**Table 1:** Optimal, minimax and admissible design for Simon’s two-stage design

***p***_**0**_	***p***_**1**_	***α***	***β***	***α***_**T**_	1-***β***_**T**_	***n***_**1**_	***r***_**1**_	***PET***_**S1**_	***n***	***r***	***EN***_**S**_	Type	***q***
0.05	0.25	0.05	0.20	0.047	0.812	9	0	0.630	17	2	12.0	Optimal	[0.000, 0.643]
				0.043	0.801	12	0	0.540	16	2	13.8	Minimax	[0.643, 1.000]
0.10	0.30	0.05	0.20	0.047	0.805	10	1	0.736	29	5	15.0	Optimal	[0.000, 0.286]
				0.040	0.806	11	1	0.697	27	5	15.8	Admissible	[0.286, 0.500]
				0.036	0.805	12	1	0.659	26	5	16.8	Admissible	[0.500, 0.730]
				0.033	0.802	15	1	0.549	25	5	19.5	Minimax	[0.730, 1.000]
0.30	0.50	0.05	0.20	0.050	0.803	15	5	0.722	46	18	23.6	Optimal	[0.000, 0.216]
				0.044	0.801	18	6	0.722	42	17	24.7	Admissible	[0.216, 0.250]
				0.046	0.804	19	6	0.666	39	16	25.7	Minimax	[0.250, 1.000]
0.05	0.25	0.10	0.10	0.093	0.903	9	0	0.630	24	2	14.6	Optimal	[0.000, 0.091]
				0.083	0.905	10	0	0.599	22	2	14.8	Admissible	[0.091, 0.333]
				0.078	0.905	11	0	0.569	21	2	15.3	Admissible	[0.333, 0.524]
				0.074	0.903	13	0	0.513	20	2	16.4	Minimax	[0.524, 1.000]
0.10	0.30	0.10	0.10	0.098	0.901	12	1	0.659	35	5	19.8	Optimal	[0.000, 0.032]
				0.10	0.904	18	2	0.734	26	4	20.1	Admissible	[0.032, 0.231]
				0.095	0.903	16	1	0.515	25	4	20.4	Minimax	[0.231, 1.000]
0.30	0.50	0.10	0.10	0.097	0.905	22	7	0.671	46	17	29.9	Optimal	[0.000, 0.111]
				0.090	0.901	21	6	0.551	42	16	30.4	Admissible	[0.111, 0.605]
				0.094	0.900	28	7	0.365	39	15	35.0	Minimax	[0.605, 1.000]
0.05	0.25	0.05	0.10	0.049	0.902	9	0	0.630	30	3	16.8	Optimal	[0.000, 0.167]
				0.047	0.913	10	0	0.599	29	3	17.6	Inadmissible	–
				0.043	0.906	10	0	0.599	28	3	17.2	Admissible	[0.167, 0.375]
				0.040	0.908	11	0	0.569	27	3	18.0	Inadmissible	–
				0.037	0.905	12	0	0.540	26	3	18.4	Admissible	[0.375, 0.667]
				0.034	0.901	15	0	0.463	25	3	20.4	Minimax	[0.667, 1.000]
0.10	0.30	0.05	0.10	0.047	0.902	18	2	0.734	35	6	22.5	Optimal	[0.000, 0.474]
				0.044	0.901	19	2	0.705	34	6	23.4	Admissible	[0.474, 0.737]
				0.041	0.902	22	2	0.620	33	6	26.2	Minimax	[0.737, 1.000]
0.30	0.50	0.05	0.10	0.050	0.903	24	8	0.725	63	24	34.7	Optimal	[0.000, 0.114]
				0.044	0.902	22	7	0.671	62	24	35.2	Inadmissible	–
				0.045	0.902	20	6	0.608	59	23	35.3	Inadmissible	–
				0.046	0.903	23	7	0.618	56	22	35.6	Admissible	[0.114, 0.250]
				0.047	0.902	24	7	0.565	53	21	36.6	Minimax	[0.250, 1.000]

(*p*_1_, *p*_0_) = (0.05, 0.25), (0.10, 0.30), (0.30, 0.50) are considered in Simon’s two-stage design. Each (*p*_1_, *p*_0_) gives three type I & II error constraints, (0.05, 0.20), (0.10, 0.10) and (0.05, 0.10), respectively. (*α*_T_, 1-*β*_T_) denotes the true type I error and power. *n*_1_ and *n* is the sample size required in the first stage and in the whole trial, respectively. (*r*_1_, *r*) are the critical values. *PET*_1_ denotes the probability of early termination at first stage. *EN* represents expected sample size

**Fig. 4:**
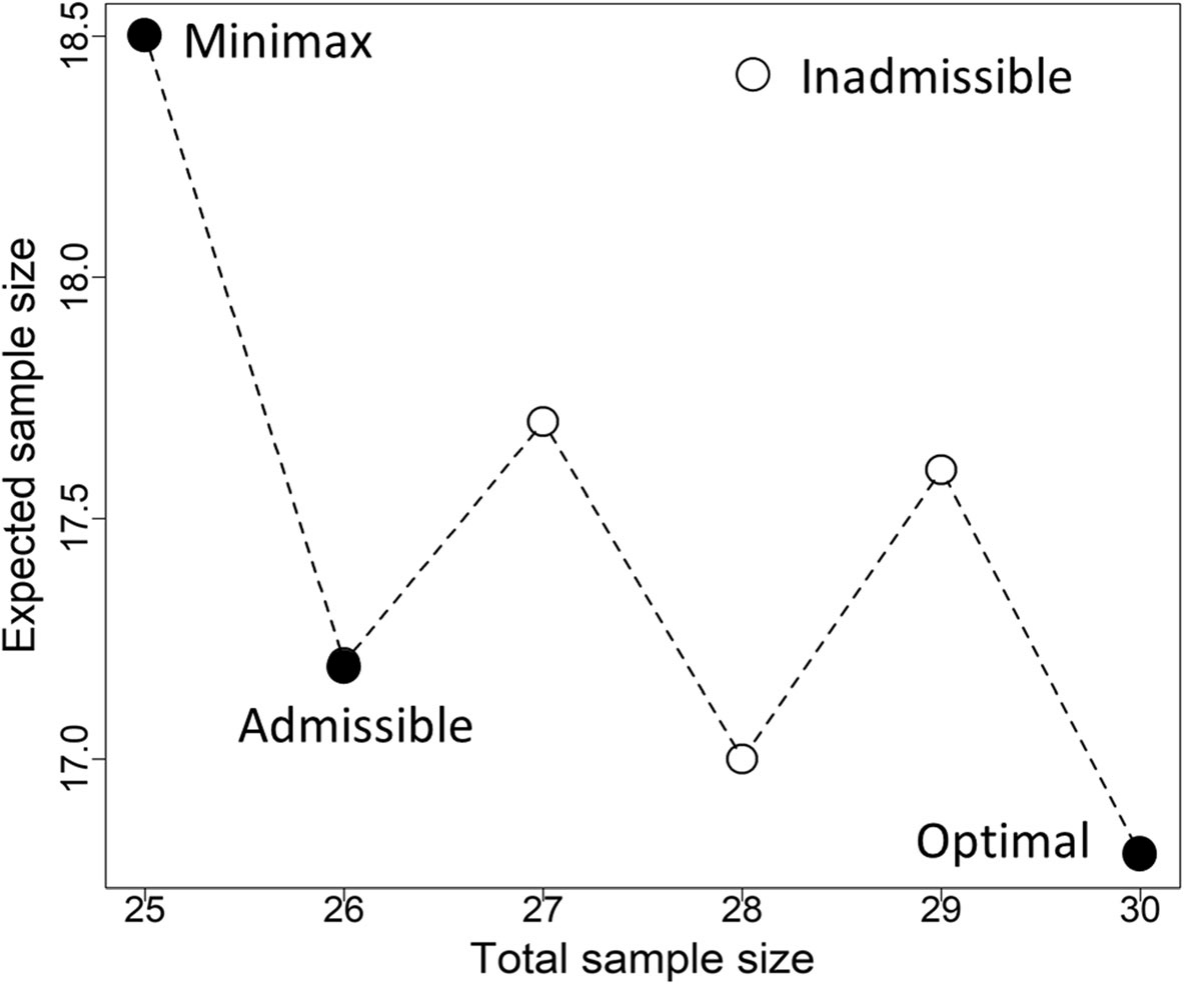
Minimax, admissible and optimal design for (*p*_0_, *p*_1_, *α*, *β*) = (0.05, 0.25, 0.05, 0.10) based on Fleming’s design

Based on Fleming’s two stage design, Table 2 displays the results of all three designs with pre-defined design parameters under *H*_0_. Similar to findings in Simon’s design, minimax design requires least *n* than that of the admissible design, and optimal design has the largest *n*. On the other hand, optimal design has the least *EN* as compare to minimax design, while admissible design provides a compromised *EN* between Fleming’s two designs. For example, when *p*_0_ = 0.05, *p*_1_ = 0.25, *α* = 0.05 and *β* = 0.10, trials will be early terminated if no more than one response is seen in the first stage. However, once > 4 responses are seen in this stage, trials will also be terminated early due to efficacy. Otherwise another 21 subjects will be enrolled and the maximum sample size becomes 30. The expected sample size is 16.8 and the probability of early termination is 0.631. One admissible designs is identified. *n* and *EN* are (26, 17.2) when *q* lies between 0.091 ~ 0.565. Figure 4 shows the expected sample sizes under *H*_0_ over a range of values for *n*. The plot starts with Fleming’s minimax design and ends with Fleming’s optimal design. Two admissible designs are highlighted in this range.

**Table 2:** Optimal, minimax and admissible design for Fleming’s two-stage design

***p***_**0**_	***p***_**1**_	***α***	***β***	***α***_**T**_	1-***β***_**T**_	***n***_**1**_	***a***_**1**_	***r***_**1**_	***PET***_**F1**_	***n***	***r***	***EN***_**F**_	Type	***q***
0.05	0.25	0.05	0.20	0.047	0.812	9	0	3	0.639	17	3	11.9	Optimal	[0.000, 0.655]
				0.043	0.801	12	0	3	0.560	16	3	13.8	Minimax	[0.655, 1.000]
0.10	0.30	0.05	0.20	0.047	0.805	10	1	5	0.738	29	6	15.0	Optimal	[0.000, 0.200]
				0.048	0.812	11	1	4	0.716	27	6	15.5	Admissible	[0.200, 0.474]
				0.049	0.817	12	1	4	0.685	26	6	16.4	Admissible	[0.474, 0.661]
				0.043	0.802	19	2	5	0.741	24	6	20.3	Minimax	[0.661, 1.000]
0.30	0.50	0.05	0.20	0.046	0.803	18	6	11	0.728	42	18	24.5	Optimal	[0.000, 0.250]
				0.049	0.807	19	6	11	0.676	39	17	25.5	Admissible	[0.250, 0.634]
				0.049	0.800	27	8	14	0.592	36	16	30.7	Minimax	[0.634, 1.000]
				0.049	0.800	21	0	12	0.009	36	16	35.9	Inadmissible	–
0.05	0.25	0.10	0.10	0.093	0.903	9	0	3	0.639	24	3	14.4	Optimal	[0.000, 0.130]
				0.083	0.905	10	0	3	0.610	22	3	14.7	Admissible	[0.130, 0.333]
				0.078	0.905	11	0	3	0.584	21	3	15.2	Admissible	[0.333, 0.500]
				0.074	0.903	13	0	3	0.538	20	3	16.2	Minimax	[0.500, 1.000]
0.10	0.30	0.10	0.10	0.085	0.900	13	1	4	0.656	31	6	19.2	Optimal	[0.000, 0.123]
				0.099	0.904	18	2	5	0.762	26	5	19.9	Admissible	[0.123, 0.231]
				0.095	0.903	16	1	5	0.532	25	5	20.2	Minimax	[0.231, 1.000]
0.30	0.50	0.10	0.10	0.097	0.901	20	6	10	0.656	47	19	29.3	Optimal	[0.000, 0.048]
				0.091	0.900	20	6	11	0.625	45	18	29.4	Admissible	[0.048, 0.117]
				0.093	0.900	23	7	12	0.640	42	17	29.8	Admissible	[0.117, 0.492]
				0.097	0.901	26	7	13	0.486	39	16	32.7	Minimax	[0.492, 1.000]
0.05	0.25	0.05	0.10	0.049	0.902	9	0	4	0.631	30	4	16.8	Optimal	[0.000, 0.091]
				0.047	0.913	10	0	4	0.600	29	4	17.6	Inadmissible	–
				0.047	0.907	10	0	3	0.610	28	4	17.0	Inadmissible	–
				0.046	0.911	11	0	3	0.584	27	4	17.7	Inadmissible	–
				0.042	0.901	11	0	3	0.584	26	4	17.2	Admissible	[0.091, 0.565]
				0.045	0.903	13	0	3	0.538	25	4	18.5	Minimax	[0.565, 1.000]
0.10	0.30	0.05	0.10	0.048	0.901	17	2	5	0.784	41	8	22.2	Optimal	[0.000, 0.032]
				0.048	0.902	18	2	6	0.740	35	7	22.4	Admissible	[0.032, 0.444]
				0.047	0.900	16	1	5	0.532	33	7	24.0	Minimax	[0.444, 1.000]
0.30	0.50	0.05	0.10	0.047	0.900	25	8	14	0.683	56	23	34.8	Optimal	[0.000, 0.250]
				0.049	0.901	26	8	14	0.637	53	22	35.8	Admissible	[0.250, 0.670]
				0.049	0.900	37	11	18	0.579	50	21	42.5	Minimax	[0.670, 1.000]
				0.05	0.900	28	0	15	0.008	50	21	49.8	Inadmissible	–

(*p*_1_, *p*_0_) = (0.05, 0.25), (0.10, 0.30), (0.30, 0.50) are considered in Fleming’s two-stage design. Each (*p*_1_, *p*_0_) gives three type I & II error constraints, (0.05, 0.20), (0.10, 0.10) and (0.05, 0.10), respectively. (*α*_T_, 1-*β*_T_) denotes the true type I error and power. *n*_1_ and *n* is the sample size required in the first stage and in the whole trial, respectively. (*a*_1_, *r*_1_, *r*) are the critical values. *PET*_1_ denotes the probability of early termination at first stage. *EN* represents expected sample size

In general, for pre-specific design parameter (*p*_0_, *p*_1_, *α*, *β*), Fleming’s two-stage design requires fewer maximum sample size and expected sample size than Simon’s. It is noteworthy that under certain criteria defined by design parameters, such as (*p*_0_, *p*_1_, *α*, *β*) = (0.05, 0.25, 0.05, 0.20), no additional admissible design can be identified. In this case, only optimal and minimax designs can routinely be considered. In this paper, parameter setting (*α*, *β*) = (0.05, 0.2) gives the most desirable sample sizes. For (*p*_0_, *p*_1_), the required *n* and *EN* remain the least in (0.05, 0.25), gradually increase in (0.10, 0.30), and attain the most in (0.30, 0.50).

## A practical example

Schiller et al. [18] published a single-arm phase II clinical trial of Axitinib for patients with advanced non-small-cell lung cancer, and objective remission rate (ORR) was used as primary endpoint to evaluate efficacy. The parameter setting (*p*_0_, *p*_1_, *α*, *β*), in this trial, were specified to be (0.05, 0.2, 0.1, 0.1).

As listed in Table 3, sample size is estimated for optimal, admissible and minimax design based on Simon’s and Fleming’s two-stage design. In Simon’s design, 12 and 37 subjects are thought to be needed in the first stage and during the whole experiment for optimal design, respectively. If no response is observed in the first stage, the trial would be early terminated due to inefficacy. The number of subjects needed for minimax design in stage I and whole trial is 18 and 32 respectively. Two admissible designs’ with compromised sample sizes lie between these two designs are also listed in the table.

**Table 3:** Comparison of three designs for (*p*_0_, *p*_1_, *α*, *β*) = (0.05, 0.2, 0.1, 0.1) based on practical example

	***n***_**1**_	***a***_**1**_	***r***_**1**_	***PET***_**1**_	***n***	***r***	***EN***	Type	***q***
Simon’s design	12	–	0	0.540	37	3	23.5	Optimal	[0.000, 0.091]
	13	–	0	0.513	35	3	23.7	Admissible	[0.091, 0.333]
	14	–	0	0.488	34	3	24.3	Inadmissible	–
	15	–	0	0.463	33	3	24.7	Admissible	[0.333, 0.630]
	18	–	0	0.397	32	3	26.4	Minimax	[0.630, 1.000]
Fleming’s design	12	0	3	0.560	37	4	23.0	Optimal	[0.000, 0.091]
	13	0	3	0.538	35	4	23.2	Admissible	[0.091, 0.268]
	16	0	3	0.483	32	4	24.3	Admissible	[0.268, 0.444]
	18	0	3	0.455	31	4	25.1	Minimax	[0.444, 1.000]

*n*_1_ and *n* is the sample size required in the first stage and in the whole trial, respectively. (*a*_1_, *r*_1_, *r*) are the critical values. *PET*_1_ denotes the probability of early termination in the first stage. *EN* represents expected sample size

In Fleming’s design, minimax design requires 18 subjects in the first stage and once one or more responses are observed after the treatment, experiment proceeds to second stage and another 13 patients will be enrolled. During the first stage, however, the trial will also be considered early termination for efficacy, if 3 or more patients’ conditions are ameliorated. At second stage, if total 4 and more positive responses are found, phase II clinical trial will be claimed to be successful and further trial will be considered. Two admissible designs are identified, with *q* ∈ [0.091, 0.268] and [0.268, 0.444], respectively. For optimal design, the number of subjects required in the first and second (if necessary) design is 12 and 25, separately. Obviously, Fleming’s designs show considerably smaller maximum sample size and expected sample size than Simon’s, given a high probability of early termination for futility as well as efficacy.

## Discussion

Simon’s two-stage design has been widely used in phase II clinical oncology trials for testing the efficacy of a single treatment regimen. The original design, however, only considers stopping for futility. Alternatively, Fleming’s design lends additional flexibility of allowing early termination by accepting the treatment regimen when initial results are extremely favorable. As a result, pharmaceutical reagents with outstanding efficacy can be early marketed, and patients can thus benefit from them. What’s more, *k-*stage (*k* ≥ 3) designs have also been proposed [8, 19, 20]. There are concerns that in practice, if the accrual is not fast, or if excessive initial failures occurs at first stage, *k*-stage designs are essentially the same as two-stage designs and will not be recommended. Thus, in this article, only two-stage design is considered. Nevertheless, further exploration is still needed in multi-stage design to ensure the successful development of effective cancer treatment.

In this paper, we compare the required sample size (*n*_1_, *n*), threshold values (*a*_1_, *r*_1_, *r*) for early termination, *EN* and the probability range (*q*) for minimax, optimal and admissible criteria in Simon and Fleming’s two-stage designs. It is often the case that maximum sample size of the optimal design is much larger than that of the minimax design, although the optimal design has the smallest expected sample size. Admissible designs are compromises between the minmax and the optimal designs. In addition, the optimal design always requires the smallest sample size in the first stage. We consider this as an important advantage of the optimal design to reduce the expected sample size as compared to other designs due to larger probability of early termination in the first stage. Thus, in clinical trial setting, optimal design may be more favorable when early data support drug ineffectiveness. This can reduce risk of exposing inactive treatments on patients, since the treatment regimen would be stopped timely once it shows low response activity. On the other hand, the minimax design required smallest maximum sample size, though this comes at the cost of larger sample size under the null hypothesis. Therefore, minimax design will be preferable if evidence agents reveals impressive therapeutic efficacy. This becomes more obvious in the consideration of the Fleming’s design. In practice, an investigator may also desire to add clinically meaningful constraint to (*p*_0_, *p*_1_) as a prior. In this case, it is better to explore in the possible ranges of *q*, and determine whether admissible design is more appropriate. Mander et al. [21] proposed a new admissible criterion by considering a more general expected loss function that includes the expected sample size under both null and alternative hypotheses and the maximum sample size. Their paper also additionally considered designs that can allow stopping for both efficacy and futility. We realized that our paper is considered as a subset of their comparisons provided no weight given to the expected sample size under alternative hypothesis. However, their triangular graph is not easily exemplifying the inadmissible designs among all candidate admissible designs. Our paper showed that the boundary line between admissible designs can still include a handful of designs that are not admissible for each set of design parameters. In addition, we are able to visually display all candidate designs between the minimax and the optimal designs in Simon’s and Fleming’s two-stage design. Our presented results further corroborated that inadmissible designs may not exist if the difference in maximum sample size between two Simon’s designs is less than or equal to 1 [22] or it is not on the concave hull [23]. Therefore, we consider both of our extensive tabulation and graphical method as important advantages to guide investigators to find the preferable design under the null hypothesis is true.

We revisit a single-arm phase II clinical trial of Axitinib for patients with advanced non-small-cell lung cancer [24]. Both optimal, minimax and admissible designs under Simon’s and Fleming’s design are used to attain 90% power at the significance level of 0.1. In this practical example, the *ENs* for three designs can be described as minimax design > admissible design > optimal design. Meanwhile, Fleming’s design always requires equal or smaller maximum sample size and expected sample size than Simon’s. This is due to the fact that Fleming’s design has the largest probability to reject further study of drugs either with novel efficacy or gloomy activity during early stage. Therefore, when accruing patients is difficult, or the study drug is costly, Fleming’s design can be a more appropriate choice. Oftentimes, two-stage design has definitive criterions for early termination, thus it can prevent subjects from continuously receiving treatment with unsatisfying efficacy. In addition, two-stage design receives popularity because of its comprehensible concept and convenient implementation. Thus, various methodological developments of this design are still expanded in many ways. For example, in some trials, even though the number of responses has exceeded threshold value *r*, the experiment will not be stopped early, but be continued to achieve enough cases for estimating confidence interval of effective rate [20].

In trials with two-stage designs, errors are inevitable no matter whether the trial is early terminated or not. If the experiment is recommended to move forward at the end of first stage, the probability of making type I error can’t be ignored (namely, false positive, meaning patients continuously take inactive drugs by error). Oppositely, type II error will be inflated once the trial is early stopped (namely, false negative, meaning patients might stop taking drugs with favorable efficacy) [25]. Obviously, the error of false negative is considered more serious because drugs may lose the chance of being further investigated once rejected. Though various designs have been put forward, more research is needed to precisely reduce the probability of false negative. For example, some oncology drug will still be presumed convincingly active despite of insufficient response rate, as long as it performs well in keeping disease stabilization. In this condition, like what Kunz and Kieser [26] have done in single-arm phase II oncology trials, we could consider using test with two binary endpoints instead of conventional one endpoint.

## Conclusion

When the (*p*_0_, *p*_1_) could be estimated accurately, it is better to explore in the range of *q*, and determine which design to choose. Optimal design is preferable on drugs with limited efficacy. Minimax design is favorable on agents with impressive efficacy. For trials whose subjects are difficult to recruit or investigated drug is relatively expensive, Fleming’s design can be a better choice, compared to Simon’s design.

## Data Availability

Not applicable.

## Abbreviations

SA-II: single-arm phase
RP-II: randomized phase II
*EN*: expected sample size
*PET*: probability of early termination
